# Experimental investigation of alternative transmission functions: Quantitative evidence for the importance of nonlinear transmission dynamics in host–parasite systems

**DOI:** 10.1111/1365-2656.12783

**Published:** 2018-01-04

**Authors:** Sarah A. Orlofske, Samuel M. Flaxman, Maxwell B. Joseph, Andy Fenton, Brett A. Melbourne, Pieter T. J. Johnson

**Affiliations:** ^1^ Department of Ecology and Evolutionary Biology University of Colorado Boulder Boulder CO USA; ^2^ Institute of Integrative Biology University of Liverpool Liverpool UK; ^3^ Department of Biology University of Wisconsin Stevens Point Trainer Natural Resources Building 446 Stevens Point WI USA

**Keywords:** behaviour, epidemiology, infectious disease, macroparasite, mathematical model, *Ribeiroia ondatrae*

## Abstract

1. Understanding pathogen transmission is crucial for predicting and managing disease. Nonetheless, experimental comparisons of alternative functional forms of transmission remain rare, and those experiments that are conducted are often not designed to test the full range of possible forms.
2. To differentiate among 10 candidate transmission functions, we used a novel experimental design in which we independently varied four factors—duration of exposure, numbers of parasites, numbers of hosts and parasite density—in laboratory infection experiments.
3. We used interactions between amphibian hosts and trematode parasites as a model system and all candidate models incorporated parasite depletion. An additional manipulation involving anaesthesia addressed the effects of host behaviour on transmission form.
4. Across all experiments, nonlinear transmission forms involving either a power law or a negative binomial function were the best‐fitting models and consistently outperformed the linear density‐dependent and density‐independent functions. By testing previously published data for two other host–macroparasite systems, we also found support for the same nonlinear transmission forms.
5. Although manipulations of parasite density are common in transmission studies, the comprehensive set of variables tested in our experiments revealed that variation in density alone was least likely to differentiate among competing transmission functions. Across host–pathogen systems, nonlinear functions may often more accurately represent transmission dynamics and thus provide more realistic predictions for infection.

## Graphical abstract

The authors tested 10 candidate functions representing the transmission of trematode parasites to amphibian hosts using laboratory experiments varying the duration of exposure, numbers of parasites, numbers of hosts and parasite density. Nonlinear functions outperformed classical frequency‐ and density‐dependent transmission functions and may provide more realistic predictions for infection.

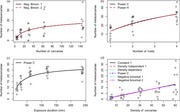

## 1 INTRODUCTION

Understanding the functional form of transmission has important implications for modelling disease impacts, forecasting future disease spread and understanding the evolution of virulence (Boots & Sasaki, 2003; De Castro & Bolker, 2005; Fenton, Fairbairn, Norman, & Hudson, 2002; Holt, Dobson, Begon, Bower, & Schauber, 2003; McCallum, Barlow, & Hone, 2001). For mathematical convenience, most models used in disease ecology characterize parasite transmission as being either density‐ or frequency‐dependent (Begon et al., 2002; Devenish‐Nelson, Richards, Harris, Soulsbury, & Stephens, 2014; Ryder, Webberley, Boots, & Knell, 2005; Smith et al., 2009). Density‐dependent transmission (typically the default option for most models of disease; McCallum et al., 2001) assumes rates of host‐to‐host contact increase linearly with population density, whereas frequency‐dependent (density‐independent) transmission (typically used to model sexually transmitted‐ and vector‐borne infections; McCallum et al., 2001) assumes a constant per‐capita rate of contact with other hosts regardless of population density (Begon et al., 2002). These models make strikingly different predictions for infection dynamics (Hoch, Fourichon, Viet, & Seegers, 2008; Smith et al., 2009; Wonham, Lewis, Rencławowicz, & van den Driessche, 2006), in particular regarding whether there is a threshold host population size for pathogen persistence (Bolker & Greenfell, 1995; Swinton, Harwood, Grenfell, & Gilligan, 1998); density‐dependent transmission predicts such a threshold, whereas frequency‐dependent transmission does not, with the consequence that parasites showing frequency‐dependent transmission can in theory drive the host population extinct (De Castro & Bolker, 2005). Given these differences, making accurate predictions about how diseases affect host population dynamics depends on identifying the appropriate transmission function, with important implications for disease management (Jennelle et al., 2014; McCallum et al., 2001; Smith et al., 2009).

Despite the ubiquity of density‐ or frequency‐dependent transmission functions in disease ecology theory, it is clear that, though mathematically convenient, these functions do not adequately capture the functional form of transmission seen in nature, and in some cases, their underlying assumptions have been proven invalid (Cross et al., 2013; Morters et al., 2013; Smith et al., 2009; Wonham et al., 2006). Given this, a range of alternative transmission functions has been proposed (McCallum et al., 2001; Table 1). Many of these functions assume that at low density contacts are likely to be directly proportional to host and parasite density (i.e. approximating density‐dependent transmission), but saturate at very high host or parasite densities (approximating frequency‐dependent transmission; Antonovics, Iwasa, & Hassell, 1995; D'Amico, Elkinton, Dwyer, Burand, & Buonaccorsi, 1996; Fenton et al., 2002; McCallum et al., 2001). Such a pattern has previously been found from analysis of infection incidence data of cowpox virus in wild rodents (Smith et al., 2009), finding that transmission was best described by a hybrid function which moved between density‐dependent transmission when host densities were low, and frequency‐dependent (density‐independent) transmission when host densities were high. A similar hybrid function has also been found to provide a better description of elk contact rates and the spread of brucellosis than either frequency or density dependence (Cross et al., 2013).

**Table 1:** The suite of transmission functions used to model transmission of the free‐living infective stages (cercariae, *C*) to encysted stages (metacercariae, *M*) of *Ribeiroia ondatrae* in Pacific chorus frog (*Pseudacris regilla*) tadpoles. Transmission is defined successful acquisition of parasite infective stages to the host over time in units of numbers of metacercariae (*M*). The form of each function used to model microparasite transmission from the literature is provided with the form used for macroparasites in this study for comparison. In the microparasite functions, *S* is the number of susceptible individuals (analogous to hosts, *H*) and *I* is the number of infectious individuals (analogous to cercariae, *C*). In all functions, β is the transmission parameter, assumed here to be constant in time. Additionally, *v* is the volume of the enclosure, *p* and *q* are the susceptible (host) and infectious (cercariae) responses that represent how densities of each independently affect transmission efficiency. Finally, *k* is the time‐dependent index of aggregation parameter for the negative binomial model. References refer to microparasite functions

Transmission form	Microparasite function	Macroparasite function	Biological interpretation of macroparasite functions
Constant Risk^1,2^ 1	β*S*	β*C* (*t*)	Rate of acquisition of parasites independent of number of hosts
Constant Risk^1,2^ 2	β*S*	β*H*	Rate of acquisition of parasites independent of number of parasites
Density‐dependent^1,3^	β*SI*	$\beta \frac{C(t)}{v}H$	Rate of acquisition of parasites depends on density of either parasites or hosts only (functions are mathematically equivalent)
Density‐independent^1,3^	$\beta \frac{\mathrm{SI}}{N}$	β*C*(*t*)*H*	Rate of acquisition of parasites depends on numbers of parasites and hosts independent of density
Ratio‐dependent^1,3^	$\beta \frac{\mathrm{SI}}{N}$	$\beta \frac{C(t)H}{(C(t)+H)v}$	Rate of acquisition of parasites depends on a ratio of contacts based on total parasite and host density
Power (in C only)^2^	β*SI* ^*q*^	β*C*(*t*)^*q*^ *H*	Rate of acquisition of parasites saturates with increasing numbers of parasites
Power (in H only)^2^	β*S* ^*p*^ I	β*C*(*t*)*H* ^*p*^	Rate of acquisition of parasites saturates with increasing numbers of hosts
Power (in both C and H)^1,2,4,5^	β*S* ^*p*^ *I* ^*q*^	β*C*(*t*)^*q*^ *H* ^*p*^	Rate of acquisition of parasites saturates with increasing numbers of both parasites and hosts
Negative binomial^1,3^ 1	$k\ln\left(1+\frac{\beta I}{k}\right)$	$k\ln\left(1+\frac{\beta C(t)}{k}\right)$	Rate of acquisition of parasites is equivalent to the negative binomial distribution of new infections among hosts encompassing heterogeneity among parasites
Negative binomial^1,3^ 2	$kS\ln\left(1+\frac{\beta I}{k}\right)$	$kH\ln\left(1+\frac{\beta C(t)}{k}\right)$	Rate of acquisition of parasites is equivalent to the negative binomial distribution of new infections among hosts encompassing heterogeneity among parasites and hosts

References: ^1^Rachowicz and Briggs (2007), ^2^Greer et al. (2008), ^3^McCallum et al. (2001), ^4^Liu et al. (1986), ^5^Hochberg (1991).

Rather than representing categorical alternatives, density and frequency dependence may capture two specific points on a wider spectrum of possible transmission shapes (McCallum et al., 2017). Reflecting this, more flexible, phenomenological functions have been proposed (e.g. power functions) that allow transmission to take a range of nonlinear forms beyond the density‐/frequency‐dependent extremes (Fenton et al., 2002; Hochberg, 1989, 1991; Liu, Levin, & Iwasa, 1986; Table 1). Mechanisms that can give rise to such nonlinear transmission functions include heterogeneities in the distribution of infectious particles, or density‐dependent mortality of the pathogen (Briggs & Godfray, 1995). However, beyond a general sense that transmission is likely to be more nonlinear than assumed by standard formulations, a comprehensive understanding of how this is driven by variation in host and parasite density or abundance is lacking (McCallum et al., 2017).

Empirically it is challenging to quantify the overall magnitude of transmission, let alone determine its functional form. Several studies have sought to do this using infection data from natural systems by embedding alternative transmission functions within larger population dynamic models and then fitting those models to observed incidence data (Begon et al., 1998, 1999; Cross et al., 2013; Rachowicz & Briggs, 2007). However, those approaches may be subject to uncertainty arising from potential inaccuracies in the specification of the wider population dynamic model or quantifying host densities, including the relevant area over which to evaluate transmission and the occurrence or timing of infection. An alternative approach is to conduct transmission experiments in the laboratory, where many of these factors can be controlled or quantified (D'Amico et al., 1996; Knell, Begon, & Thompson, 1996; Ryder, Miller, White, Knell, & Boots, 2007). Typically these experiments involve exposing different densities of uninfected (susceptible) and infected hosts (or parasite infective stages, depending on the mode of transmission), and quantifying the number of hosts that become infected over a period of time. However, the specific experimental design is crucial to differentiating among alternative transmission functions; many functions are indistinguishable in certain contexts, for example if increasing host *abundance* also increases host *density* (i.e. if arena size remains constant). Hence, distinguishing among competing transmission functions often requires independent manipulation of both host and parasite numbers and arena size (May & Anderson, 1979; McCallum et al., 2001), which surprisingly few studies have explored.

Here we experimentally tested a suite of mathematical functions as competing hypotheses to represent the transmission dynamics of a model system involving interactions between amphibian hosts and trematode parasites. Macroparasite transmission (i.e. arthropods and helminths) involves processes analogous to microparasite transmission (i.e. bacteria and viruses) with added mechanistic control often well suited for experiments. Because macroparasites often infect hosts through free‐living stages, each of which represent independent and quantifiable infection events, this system allows us to decouple the host and parasite components of transmission to help understand transmission in systems with these characteristics. Specifically, we used a maximum likelihood approach to determine which mathematical transmission function was best supported by experiments that systematically varied duration of exposure, host density, parasite density and the total number of parasites. Furthermore, we examined the role of host behaviour (via experimental manipulation using anaesthesia). Finally, we tested the generality of our conclusions by analysing two other datasets for trematode transmission (Karvonen, Paukku, Valtonen, & Hudson, 2003; Paller, Kimura, & Uga, 2007), ultimately finding that nonlinear forms of transmission were more appropriate functions than either the classic density‐dependent functions, or density‐independent and ratio‐dependent functions analogous to frequency‐dependent transmission for macroparasites.

## 2 MATERIALS AND METHODS

### 2.1 Study system

*Ribeiroia ondatrae* is a trematode in the family Echinostomatidae with a complex life cycle, sequentially infecting snails (*Helisoma trivolvis*), larval amphibians and finally amphibian‐eating birds (Johnson, Sutherland, Kinsella, & Lunde, 2004; Tkach, Kudlai, & Kostadinova, 2016). Transmission of *R. ondatrae* from snails to amphibians occurs through direct infection by free‐living aquatic parasite stages—cercariae—which then form encysted metacercariae in the amphibian that induce developmental malformations (Johnson, Lunde, Ritchie, & Launer, 1999). During the transmission process, cercariae exhibit a searching behaviour until they locate a suitable encystment site (Beaver, 1939; Johnson et al., 2004; Sutherland, 2005). Preferred encystment locations in amphibians include just below the epidermis and above the muscular tissues at the base of hindlimbs, inguinal and tail resorption area, cloaca and lower mandible (Beaver, 1939; Johnson & Hartson, 2009; Johnson et al., 2004; Sutherland, 2005). We used the Pacific chorus frog, *Pseudacris regilla*, in the family Hylidae as our focal amphibian host because it is among the species with the highest reported frequencies of malformations, approaching 90% of emerging metamorphs in some populations (Johnson, Preston, Hoverman, & Richgels, 2013). Mortality and pathology in the amphibian host is related to the intensity of infection—the total number of parasites in the host—highlighting the need for understanding transmission dynamics (Johnson et al., 2012).

### 2.2 Model development and evaluation

We compared 10 candidate models representing unique hypotheses for pathogen transmission with experimental data using an information theoretic approach following the methods of Greer, Briggs, and Collins (2008) and Rachowicz and Briggs (2007). We defined transmission in terms of successful acquisition of parasite infective stages to the host over time. We developed our set of models (Table 1) through an extensive literature search of functions used to model the spread of microparasites, macroparasites and parasitoids. We converted two variations of the canonical frequency‐dependent transmission function to forms appropriate for macroparasites, which we refer to as density‐independent and ratio‐dependent throughout. We also included three power law functions and two negative binomial functions as well as two functions representing constant risk (McCallum et al., 2001). Transmission in microparasites is typically modelled as a function of the densities of susceptible, *S,* and infective, *I*, individuals. We adapted each transmission function to apply specifically to a macroparasite system, where individual free‐living infective stages (cercariae, *C*) replace infectives (*I*). To better understand the processes underlying transmission in this system, we incorporated parasite loss from the environment via infection as one mechanism of depletion (Civitello, Pearsall, Duffy, & Hall, 2013; Civitello & Rohr, 2014). We note that successful infection removes infectives (decreasing *C*) and leads to encysted stages (metacercariae, *M*). We did not account for parasites that penetrated the host but failed to successfully infect (“irreversible contact,” Civitello et al., 2013), but previous research found that (1) the proportion of *R. ondatrae* cercariae successfully forming metacercariae was consistent across exposure levels, and (2) the loss or clearance of established metacercariae within *P. regilla* hosts is minimal over the time span of the current study (LaFonte & Johnson, 2013). The necropsy methods used here have high accuracy and precision in detecting infections, such that it is further unlikely that encysted parasites were not counted (LaFonte, Raffel, Monk, & Johnson, 2015).

Finally, our hosts (*H*) are analogous to susceptibles (*S*) in microparasite models, although because hosts can be infected by multiple parasites, there is no decrease in *H* as hosts become infected. We modelled the successful conversion of the number of free‐living infective stages (*C*) into the number of metacercariae (*M*) with the ordinary differential equation d*M/*d*t = *ϕ (*C,H*), where *C* and *M* are numbers at time *t* and ϕ is the transmission function, which may take any functional form given in Table 1. For example, ϕ *= *β*Η* represents the constant risk function for hosts, where *H* is the number of hosts and β is the transmission parameter encompassing contact between *C* and *H* and the probability of infection. We ignored other sources of cercariae mortality (Karvonen et al., 2003; Paller et al., 2007), but these were likely negligible over the short time frame of our experiments (15–240 min). We represent populations of hosts and parasites as numbers with density included explicitly, when applicable, by incorporating the volume (*v*) of the experimental tanks into the functional forms (Begon et al., 2002).

For most of the transmission functions listed in Table 1, we found the analytical solutions to each differential equation. For a subset (negative binomial 1 and 2), there were no analytical solutions, so we solved them numerically using the deSolve package (Soetaert, Petzoldt, & Setzer, 2010) in r (R Core Team, 2015). There are two different formulations for the negative binomial model used in the literature, one with hosts and one without (Briggs & Godfray,^1995^; Greer et al., 2008; Rachowicz & Briggs, 2007). For particular experimental manipulations (described below), different transmission functions reduce to equivalent forms highlighting the inability of different experimental designs to distinguish among models. For example, for three of our experimental manipulations, where the number of hosts was equal to one, the two negative binomial functions make identical predictions; however, for the host density experiment we evaluated both versions. To fit models, we assumed a binomial likelihood function for the successful conversion of a fixed number of free‐living parasites to metacercariae, where the probability of conversion was determined by the transmission model. We minimized the negative log likelihood for each model using Brent's method for single parameter models, and the Nelder–Mead method for multi‐parameter models via the optim() function in r (R Core Team, 2015). We obtained parameter estimates for each model and each experiment (Bolker, 2008). We used the corrected Akaike information criterion (AIC_c_) values to evaluate support for the different transmission functions (Burnham & Anderson, 2002; Greer et al., 2008).

### 2.3 Laboratory transmission experiments

We used a series of targeted, laboratory experiments that manipulated different variables independently with the aim of testing the alternative transmission functions (Table 1) across a range of parameter space. While other studies have investigated transmission with respect to system specific mechanisms, such as age of hosts or feeding behaviour (D'Amico et al., 1996; Goulson et al., 1995), we sought to manipulate factors relevant across host–pathogen systems and modes of transmission. We also took care to avoid confounding variables such as densities and numbers of hosts, noting that some of the most common experimental designs—i.e. varying total parasite number while keeping host and total volume constant—often do not reveal any distinction between transmission functions (see also Antonovics & Alexander, 1992; Rachowicz & Briggs, 2007).

We used *P. regilla* tadpoles raised in the laboratory from eggs and *R. ondatrae* from naturally infected snails to conduct experiments that independently varied (1) parasite number (4, 13, 30, 63 and 144 parasites), (2) host density (0.48, 0.95 and 1.9 tadpoles/L), (3) duration of exposure (15, 30, 60, 120 and 240 min) and (4) parasite density (5, 15, 30, 45 and 60 parasites/L), with each treatment replicated 10 times. For all experiments, the baseline conditions were 30 parasites/L, 30 min of exposure time and one tadpole/L. Total parasite number was manipulated independently of density by increasing volume while parasite density was held constant (30 parasites/L, range of volumes 0.12–4.8 L). Finally, our experimental design minimized heterogeneity in hosts and parasites by using tadpoles that were size‐ and stage‐matched and cercariae collected within a narrow age range (Appendices S1 and S2), thereby limiting the mechanisms that could drive nonlinear transmission dynamics.

### 2.4 Host behaviour experiment

To examine the role of host behaviour in influencing the form of transmission, we performed a fifth experiment in which we reduced host activity by anaesthesia with neutral‐buffered MS‐222 (0.125% tricaine methanesulfonate for 3 min; Appendix S3). By anaesthetizing tadpoles with a vertebrate neurotoxin, we eliminated anti‐parasite behaviours such as evasive manoeuvres, while avoiding a direct influence on trematode cercariae as supported by our observations and by previous studies (Daly & Johnson, 2011; Koprivnikar, Forbes, & Baker, 2006). Furthermore, previous research found no effect of MS‐222 on tadpole resistance to trematode infection (Sears, Snyder, & Rohr, 2013). We followed the same procedures used for varying parasite number while maintaining parasite density across container volumes (see above), with four to seven replicates per container (12 tadpoles recovered early from anaesthesia and were excluded from analysis). After each experimental exposure, we maintained tadpoles for 48 hr before quantifying parasite infection (Appendix S5). We also evaluated the effect of anaesthesia on the probability of cercarial encystment using an overdispersed binomial generalized linear model (Crawley, 2007; R Core Team, 2015).

### 2.5 Evaluation of other host–macroparasite systems

We tested the generality of our findings with *R. ondatrae* and amphibian hosts by extending our analysis to previously published data from two additional macroparasite systems: rainbow trout (*Oncorhynchus mykiss*) and the trematode *Diplostomum spathaceum* (Karvonen et al., 2003) and minnow (*Zacco temmincki*) and the trematode *Centrocestus armatus* (Paller et al., 2007). These studies experimentally exposed individual hosts to different (1) cercariae densities and (2) cercariae numbers independent of density (Appendix S5), but without any manipulation of host density or exposure duration. Although the original studies evaluated frequency‐ and density‐dependent functions, our reanalysis fit all models described in Table 1.

## 3 RESULTS

### 3.1 Overview of laboratory transmission experiments

**Figure 1:**
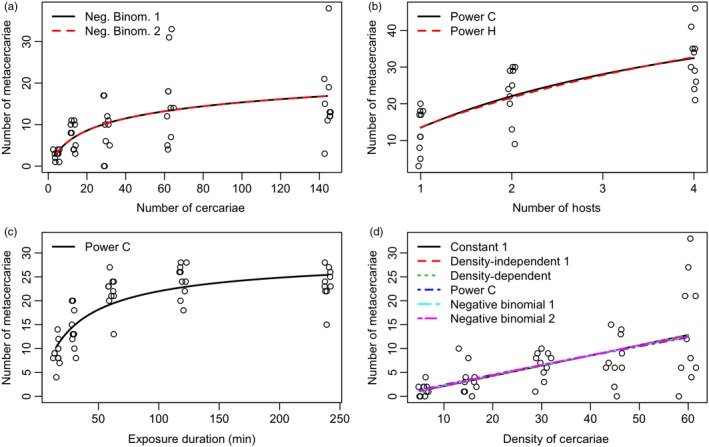
The number of *Ribeiroia ondatrae* metacercariae infecting *Pseudacris regilla* tadpoles in laboratory experiments manipulating (a) parasite number (*C*), (b) host number (*H*), (c) duration of exposure and (d) parasite density (/L) (*C*/*v*). Points in (a), (c), (d) represent the infection levels of individual tadpoles, while (b) represents the total infection level of all tadpoles in a given density treatment. Lines represent the average infection expected from the well‐supported transmission functions (within 2 Akaike information criterion (AIC) units of the best model). For some functions and experimental conditions, models reduced to the same functional form (a, d) resulting in exactly the same AIC (Table 2a–d). Note different scales of the *x*‐ and *y*‐axes. Points were jittered along the *x*‐axis to avoid overlap [Colour figure can be viewed at http://wileyonlinelibrary.com]

The manipulations produced a wide range of *R. ondatrae* metacercariae per host that scaled with variation in parasite, host or time treatments. Duration of exposure and host density had especially strong effects on transmission success (Figure 1a,b). Increases in exposure duration had a strong positive effect leading to the maximum infection level overall with *c*. 80% of cercariae successfully encysting after 240 min. Increasing host density led to an overall greater transmission success for cercariae, but individual hosts had lower average infection (metacercariae per host) when multiple tadpoles were present. With respect to differentiating among the candidate transmission functions, variation in the total number of parasites (independent of density [cercariae/L]), variation in host density and variation in the duration of exposure offered the clearest differences, while variation in parasite density alone discriminated poorly (Figure 1, Table 2). Across most experiments, we found strong support for two types of nonlinear functions, namely the power law and negative binomial functions, and poor support for classical density‐dependent, density‐independent and ratio‐dependent models (Table 2).

**Table 2:** Model selection statistics for macroparasite transmission functional forms (Table 1) according to different experimental conditions (a–e) of *Pseudacris regilla* tadpole hosts and cercariae of *Ribeiroia ondatrae*. Functional forms with identical Akaike information criterion (AIC_c_)values are mathematically equivalent under the experimental condition tested

Transmission function	β (units)	Additional parameters (units)	AIC_c_ value	ΔAIC_c_
a. Varying parasite number (constant parasite density, variable volume)				
Negative binomial 1	0.0644 (min^−1^)	*k *=* *0.1343 (min^−1^)	408.7435	0.000
Negative binomial 2	0.0644 (min^−1^)	*k* = 0.1343 (min^−1^)	408.7435	0.000
Power C	0.0810 (H^−*q*^ min^−1^)	*q* = 0.4129 (dimension‐less)	418.6477	9.904
Power CH	0.0811 (H^1−*p*−*q*^ min^−1^)	*q* = 0.4127, *p* = 3.433 (dimension‐less)	420.9141	12.171
Density‐dependent	0.0145 (H^−1^ min^−1^)		466.1725	57.429
Constant 1	0.0074 (min^−1^)		637.8375	229.094
Density‐independent	0.0074 (min^−1^)		637.8375	229.094
Power H	0.0074 (H^−*p*^ min^−1^)	*p* = 1.1254 (dimension‐less)	640.0095	231.266
Constant 2	0.1292 (min^−1^)		967.7085	558.965
Ratio‐dependent	0.1181 (min^−1^)		2164.3919	1755.648
b. Varying host density (constant volume, variable host number)				
Power C	1.0E^−5^ (H^−*q*^ min^−1^)	*q* = 2.0873 (dimension‐less)	241.7517	0.000
Power H	0.0081 (H^−p^ min^−1^)	*p* = 0.7967 (dimension‐less)	242.7335	0.982
Density‐dependent	0.0141 (H^−1^ min^−1^)		248.2491	6.497
Density‐independent	0.0067 (min^−1^)		248.2491	6.497
Power CH	6.37E^−8^ (H^1−*p*−*q*^ min^−1^)	*q* = 4.0274, *p* = 0.7766 (dimension‐less)	272.1948	30.443
Constant 2	0.3057 (min^−1^)		283.3670	41.918
Constant 1	0.0149 (min^−1^)		370.4927	128.741
Negative binomial 1	0.6214 (min^−1^)	*k* = 0.1390 (min^−1^)	372.7943	131.043
Ratio‐dependent	0.2000 (min^−1^)		1194.1242	952.372
Negative binomial 2	0.5464 (min^−1^)	*k* = 0.4679 (min^−1^)	24,677.5267	24,435.775
c. Varying duration of exposure				
Power C	3.0E^−4^ (H^−*q*^ min^−1^)	*q* = 2.3934 (dimension‐less)	301.5747	0.000
Power CH	3.0E^−4^ (H^1−*p*−*q*^ min^−1^)	*q* = 2.3956, *p* = 2.4752 (dimension‐less)	303.8412	2.266
Constant 1	0.0133 (min^−1^)		485.1008	183.526
Density‐dependent	0.0133 (H^−1^ min^−1^)		485.1008	183.526
Density‐independent	0.0133 (min^−1^)		485.1008	183.526
Power H	0.0133 (H^−*p*^ min^−1^)	*p* = 1.1294 (dimension‐less)	487.2728	185.698
Negative binomial 1	0.0133 (min^−1^)	*k* = 107,964.601 (min^−1^)	487.2731	185.698
Negative binomial 2	0.0133 (min^−1^)	*k* = 107,964.601 (min^−1^)	487.2731	185.698
Ratio‐dependent	0.1295 (min^−1^)		1,158.6684	857.094
Constant 2	0.1158 (min^−1^)		1,220.7846	919.210
d. Varying parasite density (constant volume, variable parasite number)				
Constant 1	0.0080 (min^−1^)		372.3348	0.000
Density‐independent	0.0080 (min^−1^)		372.3348	0.000
Density‐dependent	0.0080 (H^−1^ min^−1^)		372.3348	0.000
Power C	0.0114 (H^−*q*^ min^−1^)	*q* = 0.9001 (dimension‐less)	373.4308	1.096
Negative binomial 1	0.0088 (min^−1^)	*k* = 1.7293 (min^−1^)	374.2046	1.870
Negative binomial 2	0.0088 (min^−1^)	*k* = 1.7293 (min^−1^)	374.2046	1.870
Power H	0.0080 (H^−*p*^ min^−1^)	*p* = 1.1438 (dimension‐less)	374.5068	2.172
Power CH	0.0113 (*H* ^1‐*p‐q*^ min^−1^)	*q* = 0.9033, *p* = 3.2066 (dimension‐less)	375.6973	3.363
Ratio‐dependent	0.1931 (min^−1^)		548.9603	176.626
Constant 2	0.1387 (min^−1^)		639.2576	266.923
e. Host behaviour experiment				
Negative binomial 1	0.0795 (min^−1^)	*k* = 0.2449 (min^−1^)	205.8628	0.000
Negative binomial 2	0.0795 (min^−1^)	*k* = 0.2449 (min^−1^)	205.8628	0.000
Power C	0.1131 (H^−*q*^ min^−1^)	*q* = 0.4499 (dimension‐less)	217.6833	11.821
Power CH	0.1133 (H^1−*p*−*q*^ min^−1^)	*q* = 0.4500, *p* = 3.4092 (dimension‐less)	220.2804	14.418
Density‐dependent	0.0250 (H^−1^ min^−1^)		229.9331	24.070
Constant 1	0.0131 (min^−1^)		373.9949	168.132
Density‐independent	0.0131 (min^−1^)		373.9949	168.132
Power H	0.0131 (H^−*p*^ min^−1^)	*p* = 1.1506 (dimension‐less)	376.3664	170.504
Constant 2	0.1329 (min^−1^)		840.0994	634.237
Ratio‐dependent	0.1657 (min^−1^)		1183.9051	978.042

#### 3.1.1 Varying parasite number (constant parasite density, variable volume)

**Figure 2:**
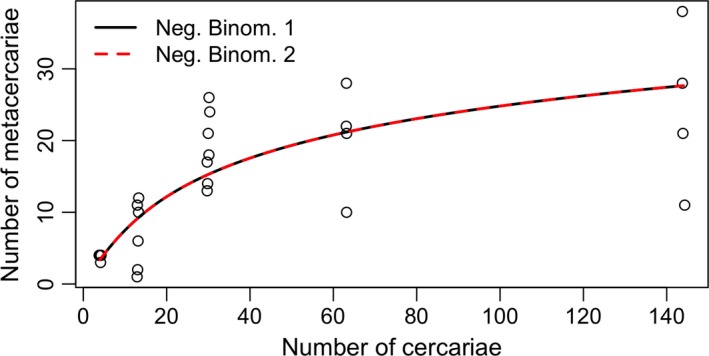
The number of *Ribeiroia ondatrae* metacercariae infecting *Pseudacris regilla* tadpoles in laboratory experiments manipulating host behaviour under conditions varying parasite number (*C*), (see text). Points represent the infection levels of individual tadpoles. Lines represent the average infection expected from the best‐fitting models (Table 2e). The two negative binomial functions under these experimental conditions reduced to the same functional form resulting in exactly the same Akaike information criterion (AIC). Points were jittered along the *x*‐axis to avoid overlap [Colour figure can be viewed at http://wileyonlinelibrary.com]

Increasing the number of parasites while keeping parasite density constant led to a nonlinear saturation of infection (Figure 1a). For instance, average infection per tadpole increased from 2.8 ± 1.1 (*SD*) metacercariae when exposed to four cercariae to 15.7 ± 9.2 (*SD*) when exposed to 144 cercariae. This relationship was best represented by the negative binomial function, and both negative binomial models were equally supported because they reduced to the same model with fixed *H* (Figure 2a, Table 2a). Overall, there was a sharp decline in the proportion of successful cercariae from 0.7 to 0.1 over the range of volumes tested (0.12–4.8 L).

#### 3.1.2 Varying host density (constant volume, variable host number)

Increases in host density led to a slightly nonlinear increase in total cercariae transmission success (Figure 1b), which increased from 37% (23.1 ± 7.3 recovered parasites of an initial 63) with a single host to 51% (32.1 ± 7.7) with four tadpoles. This was the largest increase in cercariae transmission success among all experimental manipulations; however, at the individual host level, average infection intensity declined with increases in the number of hosts as parasites became more distributed among hosts (from 23.1 ± 7.3 per host in the one tadpole condition to 8.0 ± 4.1 per host in the four tadpole condition). Both power law models (power C and power H) were well supported, with ΔAIC_c_ = 0 and 0.982 respectively (Figure 1b). Models without a host component received little support (Table 2).

#### 3.1.3 Varying duration of exposure

Increasing the duration of exposure caused a rapid increase in the proportion of successful cercariae from 0.3 to over 0.7 followed by saturation of infection at ~0.8 over exposure periods longer than 60 min (Figure 1c). This corresponded to an average infection of 8.3 ± 2.7 (*SD*) metacercariae per tadpole after 15 min rising to 21.8 ± 3.6 (*SD*) at 60 min. Based on this nonlinear saturation, the power law C model showed the greatest support, with the power law CH model next best with ΔAIC_c_ = 2.3 (Table 2c). All remaining functions underestimated the rapid transmission dynamics initially and overestimated the total infection at saturation (ΔAIC_c_ >183).

#### 3.1.4 Varying parasite density (constant volume, variable parasite number)

Finally, we observed a linear increase in average infection with increasing parasite density from 1.4 ± 1.2 (*SD*) (28% of total parasites) at an exposure of five cercariae to 13.8 ± 11.0 (*SD*) with exposure to 60 cercariae (23% of total parasites) (Figure 1d). This experiment was least able to differentiate among the competing transmission functions, providing almost equal support across all the tested functions (Table 2d). The proportion of successful cercariae remained relatively constant over the range of parasite densities from ~0.17 to 0.28.

### 3.2 Host behaviour experiment

Anaesthesia reduced tadpole activity, including anti‐parasite behaviours, leading to a 46% higher average infection success compared to treatments with unanaesthetized hosts (GLM, anaesthesia treatment *Z* = 2.2, *p *=* *.031). Regardless of whether hosts were anaesthetized, we observed a nonlinear, saturating relationship between total parasite number and infection success, such that average per‐host infection increased rapidly from ~4 to 19 before levelling off at 25 metacercariae across the range of parasite exposures. Just as with the parallel manipulation involving unmanipulated (active) hosts, this result was best represented by the negative binomial functions, though both were equally supported, again because under these conditions, they reduce to the same model (Figure 2, Table 2e). Thus, host anti‐parasite behaviours reduce net transmission, but are unlikely to be responsible for the observed nonlinear dynamics.

### 3.3 Evaluation of other host–macroparasite systems

**Figure 3:**
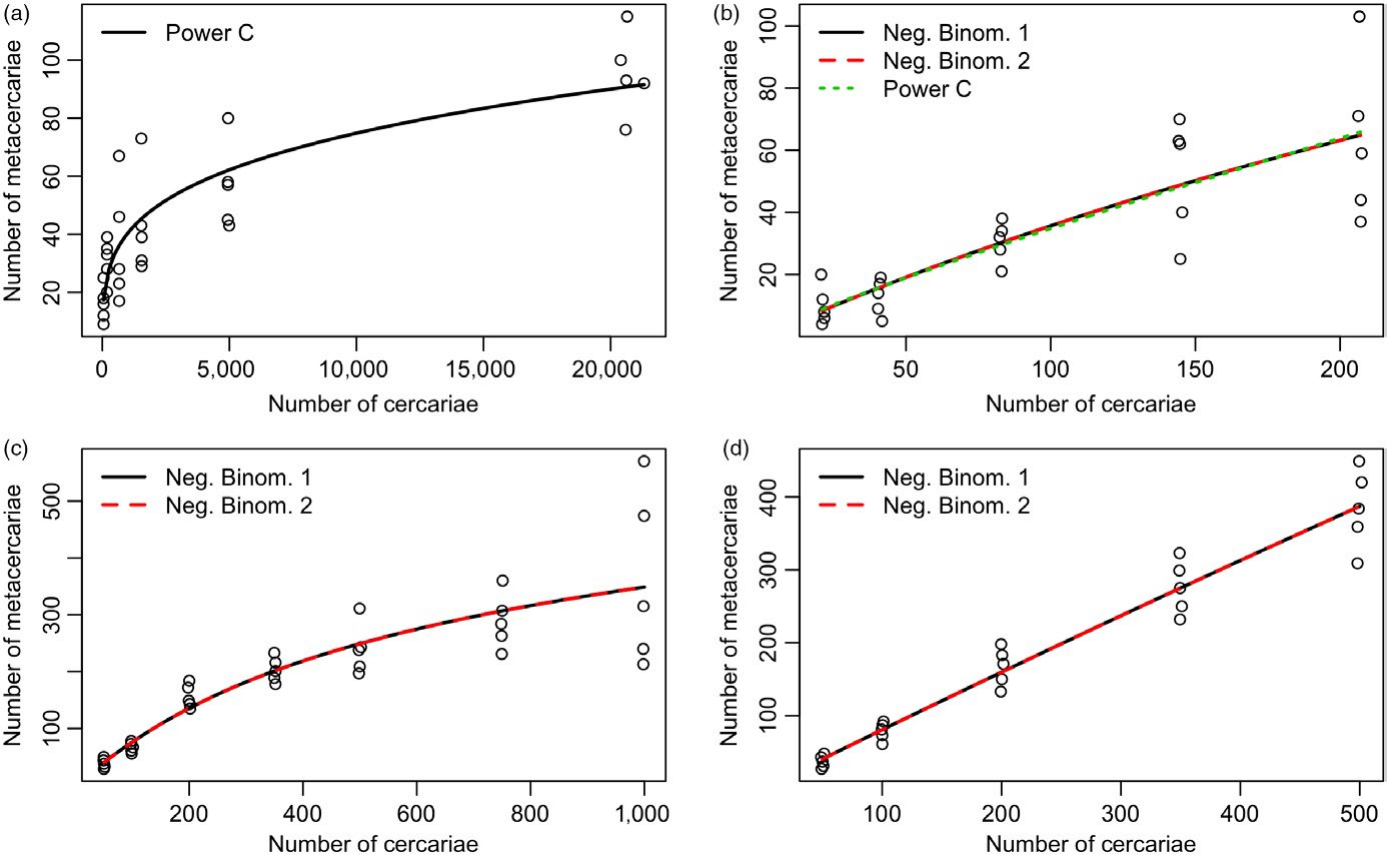
The number of *Diplostomum spathaceum* metacercariae infecting rainbow trout (*Oncorhynchus mykiss*) across a range of (a) parasite numbers (*C*), and (b) parasite density (/L) (*C*/*v*), (data from Karvonen et al., 2003). The number of *Centrocestus armatus* metacercariae attached to minnow (*Zacco temmincki*) across a range of (c) parasite numbers (*C*), and (d) parasite density (/L) (*C*/*v*), (data from Paller et al., 2007). Points represent the infection levels of individual fish. Lines represent expected values for the best‐fitting models (Table S1). For some functions and experimental conditions, models reduced to the same functional form (a, d) resulting in exactly the same Akaike information criterion (AIC). Note different scales of the *x*‐ and *y*‐axes. Points were jittered along the *x*‐axis to avoid overlap [Colour figure can be viewed at http://wileyonlinelibrary.com]

Our analysis of data from previous studies of macroparasite transmission showed congruence with our empirical results, suggesting that nonlinear transmission functions may be general across a variety of parasite and host taxa and different scales of experimental procedures (Table S1). Importantly, the results for both systems contrast the original conclusions that transmission was frequency‐dependent (density‐independent) (Figure 3; Karvonen et al., 2003; Paller et al., 2007). However, Karvonen et al. (2003) log‐transformed the data prior to analysis, which may have led to the support for a linear function for otherwise nonlinear dynamics. Similar to our empirical data, experiments manipulating parasite density were less able to distinguish between functional forms compared to when parasite numbers were varied independent of density.

For rainbow trout (*O. mykiss*) and *D. spathaceum* cercariae (Karvonen et al., 2003), there was a rapid increase in transmission followed by saturation at the highest exposure levels when the number of parasites increased independently of parasite density. This relationship supported the power C model, unlike in our experiment where the results conformed more closely with the negative binomial model (Figure 3a, Table S1). For the experiment where parasite density was varied, the negative binomial and power law C models were almost equally supported (ΔAIC_c_ 0.3–3); however, the trends appeared nearly linear similar to our experimental results from varying parasite density (Figure 3b). For minnows (*Z. temmincki*) and *C. armatus* cercariae (Paller et al., 2007), the negative binomial model was most strongly supported when parasite numbers were varied independent of parasite density, and when parasite density varied (Figure 3c,d). This function captured the apparent curvilinear relationship when parasite numbers increased and aligned closely with results of the current study involving *R. ondatrae* and amphibian hosts.

## 4 DISCUSSION

By using an array of experimental manipulations that independently varied host density, parasite density, total parasite exposure and exposure duration, we tested the efficacy of 10 different functional forms of host–parasite transmission that broadly encompass previous research from both epidemiological and ecological literatures (Table 1). We found that nonlinear transmission forms involving either a power law or negative binomial function were the best‐fitting models, consistently outperforming both density‐dependent and density‐independent functions. Thus, transmission of the trematode *R. ondatrae* to amphibian hosts within controlled exposure trials scaled in a positive but nonlinear fashion with the number of infectious parasites, the number of hosts and the duration of exposure. With the exception of the parasite density experiment where all models were nearly indistinguishable, all formulations of density‐dependent and density‐independent transmission were very poor fits to the experimental data (Table 2). By testing previously published data for two other host–macroparasite systems, we found support for the same or similar nonlinear transmission forms, suggesting such nonlinear functions may often more accurately capture host–parasite dynamics and potentially provide more realistic forecasting.

Comparing the performance of a wide variety of functional forms across five experiments with a uniquely comprehensive set of treatments offered a unique opportunity to evaluate alternative hypotheses about transmission. In particular, while parasite density is among the most commonly manipulated variables in previous studies of transmission, our results—including an analysis of previously published data—show that this is the least useful experiment for distinguishing among transmission forms. In addition, experimental manipulations, particularly of parasite density, resulted in functional forms reducing to equivalent forms with exactly the same estimated parameter values and AIC. Our experimental design achieved a wide range of infection, but some assumptions were made to establish our baseline condition, including parasite doses that would not cause direct host mortality. Nonetheless, these results highlight that combining multiple, alternative experimental designs provide greater power to distinguish among competing functions (varying parasite numbers while controlling for parasite density, varying host density and varying duration of exposure) in understanding parasite transmission dynamics.

Nonlinear transmission forms related to host and parasite densities received strong support in our analysis. Past applications of nonlinear transmission include modelling of insect pathogens (Briggs & Godfray, 1995), chytrid infection in natural populations of *Daphnia* (Johnson, Ives, Lathrop, & Carpenter, 2009) and *Schistosoma* sp. cercariae (Gao, Liu, Luo, & Xie, 2011). Mechanisms proposed to help explain nonlinear transmission dynamics include spatial aggregation in the distribution of hosts or parasites, heterogeneity in host susceptibility (immunity and physiology), and variation in the behaviour of hosts or parasitoids (Briggs & Godfray, 1995; Hochberg, 1991). Because we examined multiple predictor variables, nonlinearity can be considered in relation to any of these or could be multivariate. By considering the biology of *R. ondatrae* and amphibian hosts, several non‐mutually exclusive mechanisms may contribute to nonlinear transmission. First, because *R. ondatrae* cercariae may use chemical cues to both locate a host and encystment site once contact occurs, nonlinearity could result from differences in the probability of chemical detection with distance (Beaver, 1939; Haas, 1994). Moreover, potential interference by large numbers of simultaneously infecting cercariae may contribute to the nonlinear patterns observed in treatments that manipulated time of exposure and parasite number. Similarly, cercariae of the other trematodes considered in this study, *D. spathaceum* and *C. armatus*, both have intermittent or weak swimming behaviour to position themselves in areas that likely lead to host contact (Haas et al., 2002; Paller & Uga, 2008). This could lead to nonlinearity if the parasites that have localized in that habitat saturate and fish localize in those areas physically interacting with a subset of parasites. Second, host behavioural traits and responses to infection may contribute. Because parasites such as *R. ondatrae* cause considerable damage to the host tissue during encystment, often leading individuals with heavy infections to exhibit reduced activity 12–48 hr following exposure, shifts in host behavioural responses (including reduced avoidance behaviour) could enhance transmission in a nonlinear manner—particularly if it generated among‐host variation (Johnson & Hoverman, 2014; Preston, Boland, Hoverman, & Johnson, 2014). Other studies have reported that variation in host size or developmental stage can also influence parasite aggregation (Holland et al., 2007), although we endeavoured to keep these traits as constant as possible within our manipulations.

Identifying the specific mechanisms responsible for such nonlinearities remains a priority for disease research (e.g. Civitello & Rohr, 2014; Civitello et al., 2013). Future research should also investigate whether the form of transmission remains constant with variation in spatial scale, which would indicate that parasite behaviour remains an important driver, or if other host traits, environmental factors or host species alter the functional form of transmission under more realistic conditions. Based on our results, we suggest that transmission should be considered from both the host's perspective (e.g. how does the per‐host encounter rate with parasites vary with parasite density or number?) and the parasite's perspective (e.g. how does the per‐parasite encounter rate with hosts vary with host density?). These two perspectives are not the same, and together they combine to determine the shape of the overall transmission function, further emphasizing the need to independently vary both host and parasite density and number to capture the full interaction between them.

Despite the importance of accurately modelling transmission for forecasting disease, there are relatively few empirical studies of transmission for macroparasites, limiting opportunities to develop a generalized framework. Parasites with free‐living infective stages are often assumed to have the same transmission mode as directly transmitted pathogens, or they are not explicitly modelled because of the short time‐scale of the dynamics (May & Anderson, 1979; Rachowicz & Briggs, 2007). Omitting particular experimental conditions or only including classical forms often leads to support for frequency‐dependent transmission even when reanalysis indicated that nonlinear functions were a better fit (Karvonen et al., 2003; Paller et al., 2007). For instance, in a recent reanalysis of data involving interactions between the human blood fluke *Schistosoma mansoni* and its snail intermediate hosts, Civitello and Rohr (2014) found that mechanistic models of transmission produced nonlinear dynamics, and power law models were superior to traditional density‐dependent transmission, which did not account for parasite depletion.

Nonlinear functions lead to complex dynamics in terms of stability and the thresholds for pathogen invasion in host populations (Hochberg, 1991; Liu et al., 1986), and suggest there may also be a threshold density of infected hosts or infective stages required for disease persistence (Knell et al., 1996). Unsuitable transmission functions can therefore lead to inaccurate conclusions about the risk of pathogen‐induced extinction, the effectiveness of control strategies and the periodicity of host–pathogen cycles (De Castro & Bolker, 2005; Greer et al., 2008; Hochberg, 1989; Morters et al., 2013; Smith et al., 2009). Because of their flexibility, nonlinear forms of transmission may be more useful across a variety of host–pathogen systems in forecasting disease occurrence and number of hosts infected as opposed to strictly frequency‐ or density‐dependent transmission or when the true functional form for transmission is unknown (Fenton et al., 2002). Extending the current models to larger spatial scales and longer temporal scales will facilitate exploration of questions related to host populations and communities, including the role of changes in biodiversity, as well as the efficacy of proposed management strategies (Dobson, 2004; Johnson et al., 2013; Rudolf & Antonovics, 2005).

By considering other ecological interactions represented by nonlinear functions, transmission dynamics can be integrated with concepts from consumer resource dynamics and natural enemy ecology (Lafferty et al., 2015). In our models, the transmission coefficient is analogous to the searching efficiency of parasitoids (Knell et al., 1996). Transmission functions are also analogous to the functional response of predators (McCallum et al., 2001). This suggests that cercariae searching behaviour might be similar to parasitoids or predators (Combes, Bartoli, & Theron, 2002; Fenton & Rands, 2004). One intriguing possibility is the potential interference between cercariae for preferential encystment locations. At the short time‐scales at which transmission occurs, this mechanism may limit infection at the highest exposure levels. Further evidence for this competitive interaction between cercariae could be the increased variance in infection we observed in both the high parasite density and parasite number experiments, which have more potential for parasite interactions with variable outcomes. Our models addressed average infection, but additional experiments with larger sample sizes could help identify mechanisms for the variance and potential for parasite aggregation among hosts (Johnson & Hoverman, 2014).

## ACKNOWLEDGEMENTS

We thank R. C. Jadin for assistance with experiments and comments on earlier drafts of the manuscript and A. V. Johnson for assistance with the experiments. We are also grateful to D. M. Bortz for his help in formulating the mathematical models. We appreciate discussions with P. Hudson that aided our research ideas. We thank D. J. Civitello and an anonymous reviewer for comments helpful in improving the manuscript. This project was funded by the Society of Wetland Scientists, the University of Colorado Boulder, Department of Ecology and Evolutionary Biology, a Beverly Sears Graduate Student Grant and NSF Graduate Research Fellowships to S.A.O. and M.B.J. and an NSF grant (DEB‐0841758, DEB‐1149308) and a David and Lucile Packard Foundation Fellowship to P.T.J.J. A.F. was supported by a grant from the Natural Environmental Research Council UK (NE/N009800/1). Procedures involving vertebrate animals were approved by the University of Colorado Institutional Animal Care and Use Committee (1004.04).

## AUTHORS’ CONTRIBUTIONS

S.A.O. and P.T.J.J. devised the project and designed the research; S.A.O. conducted the literature review; S.A.O., S.M.F., M.B.J., A.F. and B.A.M. derived the mathematical models, S.A.O. performed the research; S.A.O., S.M.F., B.A.M. and M.B.J. analysed the data; S.A.O., S.M.F., M.B.J., A.F. and P.T.J.J. wrote the manuscript; and all authors edited the manuscript.

## DATA ACCESSIBILITY

r code and all experimental are available from the Dryad Digital Repository https://doi.org/10.5061/dryad.1pk42 (Orlofske et al., 2017).
