# Supplementary material for: Experimental investigation of alternative transmission functions: Quantitative evidence for the importance of nonlinear transmission dynamics in host–parasite systems
Source: J Anim Ecol. 2018 Jan 4;87(3):703–15. doi: 10.1111/1365-2656.12783 (PMC6849515; doi:10.1111/1365-2656.12783)
Supplement: Supplementary file 1 [file JANE-87-703-s001.docx]

Table S1. Model selection statistics for macroparasite transmission functional forms (table 1) according to different experimental conditions (a-b) of rainbow trout *Oncorhynchus mykiss* hosts and cercariae of *Diplostomum spathaceum* (Karvonen *et al.* 2003) and (c-d) of minnow *Zacco temmincki* and cercariae of *Centrocestus armatus* (Paller *et al.*2007). Functional forms with identical AICc values are mathematically equivalent under the experimental condition tested.

| **Transmission Function** | | **β (units)** | | **Additional Parameters (units)** | | **AICc value** | | **Δ AICc** | |
| --- | --- | --- | --- | --- | --- | --- | --- | --- | --- |
| *Parasite Density Karvonen et al. 2003* | | | | | | | | |  |
| Negative Binomial 1 | 0.0177 (min^-1^) | | k = 3.310 (min^-1^) | | 293.0772 | | 0.000 | |  |
| Negative Binomial 2 | 0.0177 (min^-1^) | | k = 3.310 (min^-1^) | | 293.0772 | | 0.000 | |  |
| Power C | 0.0275 (H^-q^ min^-1^) | | q = 0.8508 (dimension-less) | | 293.4459 | | 0.369 | |  |
| Power CH | 0.0274 (H^1-p-q^ min^-1^) | | q = 0.8513, p = 3.2283 (dimension-less) | | 296.0435 | | 2.966 | |  |
| Density Dependent | 0.0333 (H^-1^ min^-1^) | |  | | 298.9775 | | 5.900 | |  |
| Constant 1 | 0.0138 (min^-1^) | |  | | 298.9775 | | 5.900 | |  |
| Density Independent | 0.0138 (min^-1^) | |  | | 298.9775 | | 5.900 | |  |
| Power H | 0.0138 (H^-p^ min^-1^) | | p = 1.140 (dimension-less) | | 301.3490 | | 8.272 | |  |
| Constant 2 | 0.6307 (min^-1^) | |  | | 1074.2640 | | 781.187 | |  |
| Ratio Dependent | 0.2000 (min^-1^) | |  | | 3754.1331 | | 3461.056 | |  |
|  |  | |  | |  | |  | |  |
| *Parasite Number Karvonen et al. 2003* | | |  | |  | |  | |  |
| Power C | 0.2153 (H^-q^ min^-1^) | | q = 0.2661 (dimension-less) | | 297.0164 | | 0.000 | |  |
| Negative Binomial 1 | 0.0238 (min^-1^) | | k = 0.3689 (min^-1^) | | 317.5531 | | 20.537 | |  |
| Negative Binomial 2 | 0.0238 (min^-1^) | | k = 0.3689 (min^-1^) | | 317.5531 | | 20.537 | |  |
| Density Dependent | 0.0196 (H^-1^ min^-1^) | |  | | 582.7623 | | 285.746 | |  |
| Constant 2 | 1.2308 (min^-1^) | |  | | 844.4264 | | 547.410 | |  |
| Density Independent | 0.0003 (min^-1^) | |  | | 2191.3867 | | 1894.370 | |  |
| Constant 1 | 0.0003 (min^-1^) | |  | | 2191.3867 | | 1894.370 | |  |
| Power H | 0.0003 (H^-p^ min^-1^) | | p = 1.0801 (dimension-less) | | 2193.6886 | | 1896.672 | |  |
| Ratio Dependent | 1.0165 (min^-1^) | |  | | 9825.4313 | | 9528.415 | |  |
| Power CH | 1.93E^-12^ (H^1-p-q^ min^-1^) | | q = 2.9382, p = 2.2085 (dimension-less) | | 12220.0114 | | 11922.995 | |  |
|  |  | |  | |  | |  | |  |
| *Parasite Density Paller et al. 2007* | | |  | |  | |  | |  |
| Negative Binomial 1 | 0.0280 (min^-1^) | | k = 26.5653 (min^-1^) | | 560.9132 | | 0.000 | |  |
| Negative Binomial 2 | 0.0280 (min^-1^) | | k = 26.5653 (min^-1^) | | 560.9132 | | 0.000 | |  |
| Density Independent | 0.0257 (min^-1^) | |  | | 563.8305 | | 2.917 | |  |
| Density Dependent | 0.0103 (H^-1^ min^-1^) | |  | | 563.8305 | | 2.917 | |  |
| Constant 1 | 0.0257 (min^-1^) | |  | | 563.8305 | | 2.917 | |  |
| Power H | 0.0257 (H^-p^ min^-1^) | | p = 1.1357 (dimension-less) | | 566.2021 | | 5.289 | |  |
| Power CH | 0.0299 (H^1-p-q^ min^-1^) | | q = 0.9697, p = 3.1701 (dimension-less) | | 567.3284 | | 6.415 | |  |
| Power C | 6.54E^-7^ (H^-q^ min^-1^) | | q = 3.3885 (dimension-less) | | 1414.0849 | | 853.172 | |  |
| Constant 2 | 0.8215 (min^-1^) | |  | | 12554.9784 | | 11994.065 | |  |
| Ratio Dependent | 0.2000 (min^-1^) | |  | | 16645.3905 | | 16084.477 | |  |
|  |  | |  | |  | |  | |  |
| *Parasite Number Paller et al. 2007* | | |  | |  | |  | |  |
| Negative Binomial 1 | 0.0317 (min^-1^) | | k = 2.3327 (min^-1^) | | 964.0946 | | 0.000 | |  |
| Negative Binomial 2 | 0.0317 (min^-1^) | | k = 2.3327 (min^-1^) | | 964.0946 | | 0.000 | |  |
| Power CH | 0.0637 (H^1-p-q^ min^-1^) | | q = 0.6927, p = 3.2998 (dimension-less) | | 1143.6281 | | 179.534 | |  |
| Density Independent | 0.0103 (min^-1^) | |  | | 1885.7801 | | 921.686 | |  |
| Constant 1 | 0.0103 (min^-1^) | |  | | 1885.7801 | | 921.686 | |  |
| Power H | 0.0103 (H^-p^ min^-1^) | | p = 1.1184 (dimension-less) | | 1888.0339 | | 923.939 | |  |
| Density Dependent | 0.0103 (H^-1^ min^-1^) | |  | | 1976.1683 | | 1012.740 | |  |
| Power C | 8.56E^-9^ (H^-q^ min^-1^) | | q = 3.3885 (dimension-less) | | 10507.6766 | | 9543.582 | |  |
| Constant 2 | 0.8256 (min^-1^) | |  | | 14460.0594 | | 13495.965 | |  |
| Ratio Dependent | 0.1187 (min^-1^) | |  | | 41171.4253 | | 40207.331 | |  |
