# Supplementary material for: Experimental investigation of alternative transmission functions: Quantitative evidence for the importance of nonlinear transmission dynamics in host–parasite systems
Source: J Anim Ecol. 2018 Jan 4;87(3):703–15. doi: 10.1111/1365-2656.12783 (PMC6849515; doi:10.1111/1365-2656.12783)
Supplement: Supplementary file 2 [file JANE-87-703-s002.docx]

Supporting information for:

Experimental investigation of alternative transmission functions: quantitative evidence for the importance of non-linear transmission dynamics in host-parasite systems

**Table S2.** Summary of wet mass (± standard error) and developmental stages^a^ (± standard error) of Pacific Chorus frog tadpoles (*Pseudacris regilla*) used in experimental procedures to assess transmission dynamics of the trematode *Ribeiroia ondatrae*.

| **Experiment** | **Wet mass (mg) ± SE** | **Developmental Stage ± SE** |
| --- | --- | --- |
| Time | 702.0 ± 55.7 | 35.8 ± 0.4 |
| Parasite Number | 668.0 ± 67.2 | 35.8 ± 0.4 |
| Parasite Density | 661.2 ± 58.9 | 35.9 ± 0.4 |
| Host Density | 769.8 ± 86.5 | 35.2 ± 0.2 |
| Host Behavior | 640.2 ± 79.7 | 35.4 ± 0.4 |

**^a^**(Gosner 1960)

**References:**

Gosner K.L. (1960) A simplified table for staging anuran embryos and larvae with notes on identification. *Herpetologica* **16**, 183–190.
