# Supplementary material for: Experimental investigation of alternative transmission functions: Quantitative evidence for the importance of nonlinear transmission dynamics in host–parasite systems
Source: J Anim Ecol. 2018 Jan 4;87(3):703–15. doi: 10.1111/1365-2656.12783 (PMC6849515; doi:10.1111/1365-2656.12783)
Supplement: Supplementary file 3 [file JANE-87-703-s003.docx]

Supporting information for:

Experimental investigation of alternative transmission functions: quantitative evidence for the importance of non-linear transmission dynamics in host-parasite systems

*Sarah A. Orlofske*, *Samuel M. Flaxman*, *Maxwell B. Joseph, Andy Fenton, Brett A. Melbourne, Pieter T.J. Johnson*

**1. Laboratory animal collection and maintenance**

In both 2010 and 2011, we obtained Pacific chorus frog (*Pseudacris regilla*) eggs from field sites in California, USA. We maintained tadpoles in 40-L plastic containers with airstones and fed a 1:1 mixture of commercial fish food (TetraMin and Spirulina) with 50 percent water changes occurring every 2–3 days until tadpoles reached approximately stage 33 (Gosner 1960). Following experimental procedures, tadpoles were maintained in 1 L containers and fed 50:50 mixture of fish food before being euthanized and preserved.

Snail first intermediate hosts (*Helisoma trivolvis*) naturally infected with *Ribeiroia ondatrae* were collected from wetland field sites surrounding San Francisco Bay, California (Contra Costa, Alameda, Santa Clara and San Mateo counties).

**2. Experimental procedures**

For all experimental procedures, *R. ondatrae* infected snails were placed in 50-mL centrifuge tubes from 18:00 – 22:00H and to collect newly emerged cercariae. All experiments were started at 24:00H when cercariae were less than 6 hours old to minimize any differences due to cercariae age and infectivity (Karvonen *et al.* 2003; Paller *et al.* 2007). Cercariae were counted using a glass pipette under a dissecting microscope into 2-mL vials before being added to experimental containers filled with treated tap water. Exposures took place in a temperature control room at 22°C. Tadpoles were acclimated to the experimental containers for 30 minutes to allow them to resume normal activity prior to adding *R. ondatrae* cercariae. Each treatment was replicated 10 times. Experimental containers were selected to maintain the same depth (6 mm), but varied in length and width in the experiment where volumes were manipulated (range 6 X 6 mm to 42 X 29 mm).

We used ANOVA with Tukey HSD for all pairwise comparisons of tadpole wet mass (mg) and developmental stage (Gosner 1960; Table SI-3) across experiments. Tadpoles used in the experiment examining host density were significantly larger (*P* < 0.0264) than those used in all other experiments, which did not differ significantly from each other (*P* > 0.247). We found that tadpoles used in manipulations of time, parasite density and parasite number were significantly further in development than the tadpoles used in the experiment varying host density (*P* < 0.001). Likewise tadpoles in parasite density and number experiments were significantly further in development than those used in the manipulation of host behavior (*P* < 0.0175). However, the mean developmental stage across each experiment only varied from 35.2 to 35.9, less than one stage, so we believe that stage was maintained consistently across experiments.

**3. Amphibian anesthesia**

We used a dilute solution of MS-222 to reduce the role of tadpole anti-parasite behavior, thus isolating the role of parasite behavior in transmission dynamics. Our methods followed Daly & Johnson (2010) and were effective in removing anti-parasite behaviors of the tadpoles for approximately 25–30 minutes during exposure. The numbers of replicates for the different volume treatments varied from 4–7 depending on the numbers of tadpoles that remained anesthetized for the required 25–30 minutes or did not recover from anesthesia.

**4. Amphibian necropsy**

Tadpoles were euthanized with buffered MS-222 (Tricaine methanesulfonate, Western Chemical Inc.) and preserved in 10% buffered formalin until necropsy, where we inspected all external surfaces and removed and examined all muscle tissue and organs using a dissecting microscope. Metacercariae were examined under a compound microscope to observe distinguishing features to allow for species identification (Schell *et al.* 1985; Johnson & McKenzie 2009; Szuroczki & Richardson 2009).

**5. Previous empirical data**

We collected data representing the number of parasites infected or attached to hosts based on experiments varying parasite density and parasite number using Plot Digitizer 2.5 (Karvonen *et al.* 2003; Fig. 4a, b; Paller *et al.* 2007; Fig. 3a, b). If a point was not distinguishable from other points because they overlapped those points were not included in the analyses.

**References:**

Daly E.W. & Johnson P.T.J. (2011) Beyond immunity: quantifying the effects of host

anti-parasite behavior on transmission. *Oecologia* **165**, 1043–1050.

Gosner K.L. (1960) A simplified table for staging anuran embryos and larvae with notes

on identification. *Herpetologica* **16**, 183–190.

Johnson P.T.J. & McKenzie V.J. (2009) Effects of environmental change on helminths

infections in amphibians: exploring the emergence of *Ribeiroia* and *Echinostoma*

infections in North America. In: *The Biology of Echinostomes* (eds. Fried B,

Toledo R.). Springer, New York, pp. 249–280.

Karvonen A., Paukku S., Valtonen E.T. & Hudson P.J. (2003) Transmission, infectivity

and survival of *Diplostomum spathaceum* cercariae. *Parasitology* **127**, 217–224.

Paller V.G.V., Kimura D. & Uga S. (2007) Infection dynamics of *Centrocestus armatus*

cercariae (Digenea: Heterophyidae) to second intermediate fish hosts. *Journal of*

*Parasitology* **93**, 436–439.

Schell S.C. (1985) *Handbook of trematodes of North America north of Mexico*.

University press of Idaho.

Szuroczki D. & Richardson J.M.L. (2009) The role of trematode parasites in larval

anuran communities: an aquatic ecologist’s guide to the major players. *Oecologia*

**161**, 371–385.
